# CXCL5 polymorphisms are associated with variable blood pressure in cardiovascular disease-free adults

**DOI:** 10.1186/1479-7364-6-9

**Published:** 2012-08-02

**Authors:** Amber L Beitelshees, Christina L Aquilante, Hooman Allayee, Taimour Y Langaee, Gregory J Welder, Richard S Schofield, Issam Zineh

**Affiliations:** 1 Division of Endocrinology, Diabetes and Nutrition, University of Maryland School of Medicine, 660 W. Redwood St, HH469, Baltimore, MD, 21201, USA; 2 Department of Pharmaceutical Sciences, University of Colorado Skaggs School of Pharmacy and Pharmaceutical Sciences, Aurora, CO, 80045, USA; 3 Department of Preventive Medicine and Institute for Genetic Medicine, Keck School of Medicine, University of Southern California, Los Angeles, CA, 90089, USA; 4 Department of Pharmacotherapy and Translational Research, Center for Pharmacogenomics, University of Florida College of Pharmacy, Gainesville, FL, 32610, USA; 5 Division of Cardiovascular Medicine and Department of Veterans Affairs Medical Center, University of Florida College of Medicine, Gainesville, FL, 32603, USA

**Keywords:** CXCL5, ENA-78, Blood pressure, Hypertension, Leukocytes

## Abstract

**Objective:**

Leukocyte count has been associated with blood pressure, hypertension, and hypertensive complications. We hypothesized that polymorphisms in the *CXCL5* gene, which encodes the neutrophilic chemokine ENA-78, are associated with blood pressure in cardiovascular disease (CVD)-free adults and that these polymorphisms are functional.

**Methods and results:**

A total of 192 community-dwelling participants without CVD or risk equivalents were enrolled. Two *CXCL5* polymorphisms (−156 G > C (rs352046) and 398 G > A (rs425535)) were tested for associations with blood pressure. Allele-specific mRNA expression in leukocytes was also measured to determine whether heterozygosity was associated with allelic expression imbalance. In −156 C variant carriers, systolic blood pressure (SBP) was 7 mmHg higher than in −156 G/G wild-type homozygotes (131 ± 17 vs. 124 ± 14 mmHg; *P* = 0.008). Similarly, diastolic blood pressure (DBP) was 4 mmHg higher in −156 C variant carriers (78 ± 11 vs. 74 ± 11 mmHg; *P* = 0.013). In multivariate analysis of SBP, age, sex, body mass index, and the −156 G > C polymorphism were identified as significant variables. Age, sex, and the −156 G > C SNP were further associated with DBP, along with white blood cells. Allelic expression imbalance and significantly higher circulating ENA-78 concentrations were noted for variant carriers.

**Conclusion:**

*CXCL5* gene polymorphisms are functional and associated with variable blood pressure in CVD-free individuals. The role of *CXCL5* as a hypertension- and CVD-susceptibility gene should be further explored.

## Introduction

The relationship between inflammation and elevated blood pressure is increasingly being evaluated [1,2]. It has been shown that elevated concentrations of prototypical pro-inflammatory markers such as interleukin-6, C-reactive protein (CRP), and tumor necrosis factor-alpha are associated with increased blood pressure, incidence of hypertension, and the likelihood for hypertensive complications [3-14]. It has been further suggested that this inflammatory-hypertensive relationship results from increased number or activity of common cellular mediators such as white blood cells (WBC) [15,16]. For example, studies have demonstrated elevated WBC count to be associated with increased incident hypertension as well as increased blood pressure within the normal to pre-hypertensive range [17-22].

Although the exact mechanistic relationship between leukocytosis and elevated blood pressure is unknown, it is plausible that low-grade inflammation may be a contributing factor. In this regard, WBC count may be a surrogate marker for increased activation of inflammatory pathways that cause leukocyte recruitment and activation. As such, increased activity of leukocytic chemokines could be related to increased blood pressure.

Epithelial neutrophil activator-78 (ENA-78), a key leukocytic chemokine that is both a neutrophil attractor and activator, has been implicated in many diseases with an inflammatory component (e.g., obesity, diabetes, subclinical atherosclerosis, acute coronary syndromes) [23-32]. We have previously reported that two single nucleotide polymorphisms (SNPs), -156 G > C (rs352046) and 398 G > A (rs425535), in the gene encoding ENA-78 (*CXCL5)* occur in sites important for transcription and exon splicing [33]. In our previous work, a relationship existed between these SNPs and both plasma concentrations and leukocyte production of the ENA-78 chemokine protein [33]. We then went on to show an association between the *CXCL5* -156 G > C polymorphism and worse outcomes in patients with acute coronary syndromes [27]. In the present work, to the extent that ENA-78 is important in neutrophil recruitment and degranulation, we hypothesized that one or both of these polymorphisms (−156 G > C and 398 G > A) could be associated with differences in blood pressure in individuals without established cardiovascular disease (CVD). Specifically, we hypothesized that relatively young individuals without known CVD who were carriers of *CXCL5* variant alleles would exhibit higher systolic blood pressure (SBP), diastolic blood pressure (DBP), or pulse pressure (PP) than wild-type homozygotes. Furthermore, to assess whether there was a functional role for these polymorphisms, we measured allele-specific mRNA expression of *CXCL5* in leukocytes obtained from CVD-free individuals who were heterozygous for the SNPs at both loci.

## Materials and methods

### Study population

The study population has been previously described [33]. Briefly, participants were recruited from two sites in the USA and had to be at least 18 years of age without known CVD or CVD-risk equivalents (e.g., diabetes, peripheral vascular disease, 10-year Framingham Risk ≥20%) as defined by National Cholesterol Education Program criteria [34]. Other exclusions were pregnancy, malignancy, substance abuse, and routine use of medications known to affect WBC counts such as systemic steroids and other anti-inflammatory agents. Individuals were excluded from analysis if they were taking anti-hypertensive medications for either cardiovascular or non-cardiovascular indications (e.g., migraine). For blood pressure measurement, subjects were seated for at least 5 min in a quiet, temperature-controlled General Clinical Research Center (GCRC) outpatient clinic room, and two blood pressure measurements were taken at least 5 min apart. The average of the duplicate blood pressure measurements was used for this investigation. Blood samples were obtained from participants enrolled in University of Florida- and Colorado Multiple Institutional Review Board (IRB)-approved studies. All subjects provided written informed consent to specimen and data use in genetic association and related studies.

### Genotype and inflammatory biomarker determination

Genomic DNA was isolated from whole blood or buccal cells using previously described methods [35]. *CXCL5* genotypes were determined by polymerase chain reaction (PCR) and pyrosequencing (Qiagen, Valencia, CA, USA) as we have previously described [36]. Circulating high-sensitivity CRP (as a non-specific marker of inflammation) was measured by the Shands Hospital Laboratory at the University of Florida and University of Colorado GCRC. ENA-78 concentrations were measured by cytometric fluorescence detection as previously described (Luminex™100 IS system; Luminex Corp., Austin, TX, USA; Fluorokine® MAP Multiplex Human Cytokine Panel A; R&D Systems, Minneapolis, MN, USA) [37]. Samples were stored at −80°C until CRP and ENA-78 detection was performed.

### Allele-specific mRNA quantification

To determine whether variant carrier status results in functional changes at the transcriptional level, we quantified allele-specific mRNA transcripts from leukocytes using pyrosequencing-based methodology [38,39]. Specifically, the presence or absence of allelic expression imbalance was determined using leukocytes obtained from 18 individuals who were heterozygotic for both the −156 G > C and 398 G > A polymorphisms. The 398 G > A SNP was chosen as the genetic biomarker in these experiments because it is located in the coding region of *CXCL5*, while −156 G > C is a promoter polymorphism and as such cannot be quantified at the mRNA level. Because of the near complete linkage of the studied SNPs, we chose individuals who were heterozygotes at both loci so that 398 G > A genotype might serve as a functional surrogate for the upstream promoter locus.

Leukocyte mRNA was prepared from approximately 6 × 10^6^ cells from each individual using the RNeasy mini kit (Qiagen, Valencia, CA, USA). Cells were rinsed, lysed, and homogenized in buffered solutions and subsequently passed through the RNeasy mini column (Qiagen, Valencia, CA, USA). Following a series of washes at room temperature and 15-min incubation with DNase, concentrations were determined by spectrophotometry (NanoDrop Technologies, Wilmington, DE, USA). cDNA was synthesized using approximately 450 ng of cellular RNA from each individual using a High-Capacity cDNA Archive Kit (Applied Biosystems, Foster City, CA, USA) per protocol. Conditions for reverse transcription were 25°C for 10 min followed by 37°C for 2 h. cDNA quality was assessed by comparing cDNA and DNA PCR products generated using intron-spanning primers by gel electrophoresis. For allele-specific transcript quantification, subject DNA and cDNA underwent PCR simultaneously using previously described conditions [36]. PCR products obtained for genotype determination (DNA) and transcript quantification (mRNA) were assayed in parallel pyrosequencing reactions to minimize cycle variability. Pyrosequencing analyses were performed in duplicate on three separate PCR amplification products, and the results were pooled for analysis. Peak heights were determined by the pyrosequencing allele quantification algorithm. In genomic DNA, the ratio of 398A:G alleles for DNA in heterozygotes is expected to be approximately 1, whereas significant deviations from this ratio in mRNA would suggest allele expression imbalance associated with the variant allele.

### Statistical analyses

Genotype frequencies were determined by allele counting, and departures from Hardy-Weinberg equilibrium were assessed by chi-square analyses. Differences in blood pressure by genotype groups (0, homozygous for common allele; 1, heterozygous or homozygous for variant allele) were compared using one-way ANOVA. Based on the preexisting sample size and prevalence of variant alleles, we had 80% power with a two-sided α of 0.05 to detect a 6-mmHg difference in SBP, 4-mmHg difference in DBP, and 4-mmHg difference in PP between genotype groups. Multiple regression analysis was performed if blood pressure differences were seen across genotype groups. Covariates for multiple regression were chosen through univariate analyses of age, sex, smoking status (0, non-smoker; 1, current smoker), body mass index (BMI), CRP concentration, ENA-78 concentration, and WBC count. Any variable with a *P* ≤ 0.1 on univariate analysis was entered into the multivariable model. Because of small numbers of individuals within racial groups, analyses could not be performed within racial strata. However, race (0, white; 1, non-white) was included in all multivariable analyses, and a race-by-genotype interaction term was considered in the regression models to avoid spurious associations secondary to racial differences in allele frequency. Multiple regression using step-type selection methods was performed to determine the joint effects of *CXCL5* genotypes and clinical variables on SBP, DBP, or PP. All statistical analyses were performed using SPSS (version 11.5, SPSS Inc., Chicago, IL, USA) or SAS (version 9.1, SAS Institute Inc., Cary, NC, USA). A *P* value < 0.05 was considered statistically significant.

## Results

Baseline demographic characteristics are shown in Table 1. Participants were on average 39 ± 12 years old with blood pressures of 126/75 ± 15/11 mm Hg. -156 G > C and 398 G > A genotypes were determined for 189 and 188 of the 192 individuals, respectively. The overall −156 C and 398A minor allele frequencies were both 15%. Variant allele frequencies differed by race whereby the −156 C allele frequency was 14%, 45%, and 11%, and 398A allele frequency was 13%, 46%, and 9% in Caucasians, blacks, and non-black Hispanics, respectively. Genotype distributions satisfied criteria for Hardy-Weinberg equilibrium (data not shown). The two SNPs were in a high degree of linkage disequilibrium with *r*^2^ for Caucasian, black, and Hispanic individuals of 0.82, 1.0, and 0.51, respectively, in our study population.

**Table 1:** Baseline characteristics

**Characteristic**	***N*** **= 192**
Age (mean ± SD, years)	39 ± 12
Women (number (%))	124 (65)
Race/ethnicity (number (%))	
White	148 (77)
Black	12 (6)
Hispanic	19 (10)
Other	13 (7)
Family heart disease history (number (%))	29 (15.1)
Smoking (number (%))	35 (18)
Body mass index (mean ± SD, kg/m^2^)	29.6 ± 7
Blood pressure (mean ± SD, mmHg)	
Systolic	126 ± 15
Diastolic	75 ± 11
Pulse pressure (mean ± SD, mmHg)	51 ± 10
Cholesterol (mean ± SD, mg/dL^a^)	
Total	201 ± 43
LDL	118 ± 36
HDL	55 ± 17
Triglycerides	139 ± 107
White blood cell count, (mean ± SD, ×10^9^ cells/L)	6.3 ± 2.0
C-reactive protein (median (range), mg/L^b^)	1.78 (0.1–16.9)
ENA-78 (median (range), pg/mL^b^)	362 (32.2–3970)

^a^Total, HDL, and triglycerides available in 94% of subjects; LDL available in 92% of subjects. ^b^CRP and ENA-78 available for 88% and 91% of subjects, respectively.

### Genotype association with blood pressure

In −156 C variant carriers, SBP was 7-mmHg higher than in −156 G/G wild-type homozygotes (131 ± 17 vs. 124 ± 14 mmHg; *P* = 0.008). Similarly, DBP was 4-mmHg higher in −156 C variant carriers (78 ± 11 vs. 74 ± 11 mmHg; *P* = 0.013). PP did not differ between −156 C variant carriers and wild-type homozygotes (53 ± 11 vs. 51 ± 10; *P* = 0.22). Because of the high degree of linkage disequilibrium between the 398 G > A and −156 G > C SNPs, blood pressure differences were similar when compared by 398 G > A genotypes. For example, SBP was 130 ± 16 and 125 ± 14 mmHg in 398A variant carriers and 398 G/G homozygotes, respectively (*P* = 0.033); DBP was 78 ± 11 and 74 ± 11 mmHg, respectively (*P* = 0.038); and PP was not different between groups (53 ± 11 vs. 51 ± 10 mmHg in 398A carriers and 398 G/G homozygotes, respectively; *P* = 0.362).

**Figure 1:**
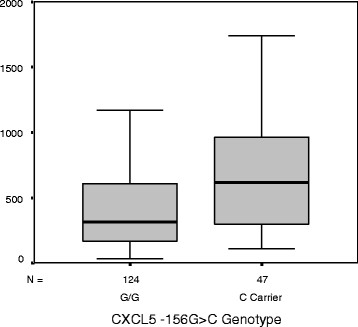
**Plasma ENA-78 by *CXCL5 *-156 G > C genotype. ***P* = 0.003; data were similar for the exon 2 SNP, data not shown (*P* = 0.001).

Age (*P* ≤ 0.001), sex (*P* ≤ 0.008), and BMI (*P* ≤ 0.002) were common univariate predictors of SBP, DBP, and PP. Furthermore, WBC count (*P* = 0.10 for SBP; *P* = 0.076 for DBP) and both *CXCL5* polymorphisms (range *P* = 0.008 to 0.038) were additional predictors of SBP and DBP, while smoking status was associated with SBP alone (*P* = 0.038). In terms of circulating CRP and ENA-78 levels, both biomarkers were significant for SBP (*P* = 0.005 for CRP and *P* = 0.033 for ENA-78) and PP (*P* = 0.001 for CRP and *P* = 0.007 for ENA-78) in univariate analyses. Consistent with our previous report, *CXCL5* genotype was associated with ENA-78 protein concentrations in the plasma whereby variant carriers at either SNP locus had higher protein concentrations than wild-type homozygotes (*P* = 0.003; Figure 1).

In multivariate analysis of SBP, age, sex, BMI, and the *CXCL5* -156 G > C promoter polymorphism were identified as significant variables (Table 2). The overall model that included these variables explained 32.5% of the variability in SBP (*P* < 0.001). Consideration of the 398 G > A polymorphism rather than the −156 G > C promoter SNP resulted in a model in which only age, sex, and BMI were significantly associated with SBP (*R*^2^ = 0.301; *P* < 0.001).

**Table 2:** Multivariate predictors of systolic blood pressure in cardiovascular disease-free individuals

**Variable**	***β***	**Standard error**	***P*value**
Constant	100	4.86	<0.0001
Age	0.313	0.094	0.001
Sex	−9.84	2.12	<0.0001
BMI	0.637	0.160	<0.0001
−156 C carrier	4.93	2.30	0.034

*R*^2^ = 0.325; *P* < 0.0001.

Age, sex, and the −156 G > C SNP were further associated with DBP, along with WBC (Table 3). Consideration of this promoter SNP (model *R*^2^ = 0.168; *P* < 0.0001) was slightly more informative than consideration of the 398 G > A SNP (*P* = 0.067) in which case age (*P* < 0.0001), sex (*P* = 0.001), and WBC (*P* = 0.02) still remained significant (model *R*^2^ = 0.145; *P* < 0.0001). In multivariable models of PP, only sex (*P* < 0.004) and BMI (*P* < 0.0001) were significant (model *R*^2^ = 0.247; *P* < 0.0001).

**Table 3:** Multivariate predictors of diastolic blood pressure in cardiovascular disease-free individuals

**Variable**	***β***	**Standard error**	***P*value**
Constant	63.13	3.42	<0.0001
Age	0.247	0.063	<0.0001
Sex	−5.801	1.549	<0.0001
−156 C carrier	3.735	1.630	0.023
WBC	0.768	0.374	0.041

*R*^2^ = 0.168; *P* < 0.0001.

### Allelic expression imbalance

**Figure 2:**
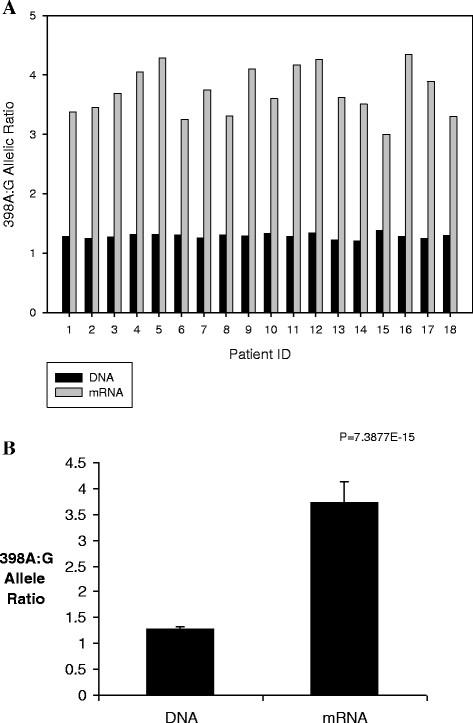
**Allele-specific *CXCL5 *mRNA expression in leukocytes.** (**A**) Allelic mRNA and DNA ratios were measured in 18 cardiovascular disease-free individuals heterozygous for the 398 G > A SNP. The A/G ratios in DNA were close to 1 suggesting equal abundance of both alleles, whereas there was consistently higher expression of mRNA from the 398A allele compared to the 398 G allele. (**B**) Pooled 398A/G ratios from 18 heterozygous individuals. The sample displayed 2.9-fold higher expression of 398A variant transcripts compared to the 398 G allele (*P* = 7.4E-15). Data are presented as mean ± SD

Allele-specific mRNA quantification was performed to determine whether there is a functional basis for the differences seen in blood pressure based on *CXCL5* genotypes (see ‘Materials and methods’ section for rationale of 398 G > A as marker SNP). Importantly, there was consistently higher expression of *CXCL5* mRNA from the 398A allele compared to the 398 G allele in heterozygous individuals (Figure 2A). For example, individual heterozygotes displayed anywhere from 2.2-fold to 3.4-fold higher expression of 398A variant transcripts compared to the 398 G allele, with a mean ratio of 2.9 (Figure 2B; *P* = 7.4E-15).

## Discussion

Accumulating evidence points to a relationship between inflammation and blood pressure. Data suggest that WBC counts are associated with incident hypertension and correlated with blood pressure concentrations. We hypothesized that WBC count is a surrogate for leukocytic chemokine activity and that the *CXCL5* gene, which encodes the neutrophil attractor ENA-78, may be an important determinant of blood pressure. We demonstrated a significant, independent relationship between *CXCL5* polymorphisms and SBP and DBP in the overall population of CVD-free individuals. Variant carriers of the −156 G > C promoter SNP had 7-mmHg and 4-mmHg higher SBP and DBP, respectively, than those with the wild-type −156 G/G genotype. Because of the epidemiologically significant difference in CVD risk conferred by blood pressure differences of this magnitude, and since variant carriers represent approximately 30% of the population studied, *CXCL5* polymorphisms should be considered as a potential novel biomarker of pre-hypertension, hypertension, and CVD risk requiring future study. However, it is important to emphasize that genetic associations are preliminary and will require confirmation in additional populations.

Of particular interest, WBC count (along with traditional variables such as age, sex, smoking status, and BMI) was significantly associated with SBP and DBP in univariate analysis among CVD-free individuals. This finding supports the report by Orakzai et al. that demonstrated a relationship between WBC counts and SBP among nearly 3,500 white individuals without CVD and with SBP < 140 mmHg on entry [20]. It also supports data from other clinical cohorts showing an association between WBC count, major WBC components (e.g., neutrophils), and blood pressure [21,22,40,41]. However, in our analysis WBC count was no longer a significant predictor of SBP when *CXCL5* genotype was included in multivariable analysis, suggesting genotype may capture the contribution of inflammation to SBP more effectively than WBC count. WBC did, however, remain a significant predictor of DBP in multivariate analysis, along with age, sex, and *CXCL5* -156 G > C genotype.

To determine whether there is any functional basis for an observed association between *CXCL5* variant alleles and blood pressure, we performed allele expression imbalance experiments in a subset of participants. The exonic 398 G > A allele was chosen as the genetic marker given its location in the coding region of the mRNA. However, the 398 G/A heterozygous individuals (*N* = 18) were also heterozygous for the promoter polymorphism, which minimizes confounding of an association by differing genotypes at the upstream locus. It was noted that variant carriers displayed nearly threefold higher expression of variant *CXCL5* mRNA transcripts from the 398A allele. This novel finding is consistent with our previous observation that variant carriers exhibited higher plasma and leukocyte-produced ENA-78 than wild-type homozygotes and that the promoter and exonic SNPs occur in transcription factor binding and splicing enhancer sites, respectively [33]. Given that the −156 G > C and 398 G > A SNPs are in near perfect linkage disequilibrium, it is unclear which polymorphism is the causal variant and functionally contributes to the blood pressure phenotype. However, the −156 G > C promoter SNP was more significantly correlated with blood pressure in our study. Further functional studies of these SNPs are warranted.

In addition to genotype and traditional covariates, we included plasma CRP and ENA-78 protein concentrations in our analyses. While CRP and ENA-78 were significantly associated with SBP (and PP) in univariate analyses, they fell out of the models when *CXCL5* genotype was included. This suggests that in our analyses, genotype is more significantly associated with the blood pressure phenotype than systemically circulating concentrations of the non-specific inflammatory mediator CRP and the *CXCL5* protein product ENA-78. While this observation may appear somewhat contradictory, it can be postulated that *CXCL5* gene polymorphisms may be better indicators of chemokine activity at the target organ (e.g., endothelium) level than a measurement in the circulation. Because of trans-acting influences on systemic biomarker expression, polymorphisms in *CXCL5* may be more robustly associated with blood pressure. In fact, we have shown a similar finding in a different population for the endothelial nitric oxide synthase gene where *NOS3* gene polymorphisms, but not measures of circulating NO activity, were associated with arterial stiffness in children with type 1 diabetes [42,43]. Further support for this observation can be found in a case–control study of the role of ENA-78 in patients with ischemic stroke. Zaremba et al. demonstrated that serum ENA-78 protein concentrations were not different between stroke patients and controls; contrarily, it was demonstrated that ENA-78 concentrations were significantly higher (twofold) in the cerebrospinal fluid of stroke patients compared with controls [44]. Taken in sum, it is possible that genotype more effectively captures the likelihood for local preponderance of chemokine activity than plasma protein level.

In general, there is biological plausibility for the role of *CXCL5* in CVD. For example, the protein product of *CXCL5*, ENA-78, belongs to the same class of chemokines as IL-8, IP-10, and I-TAC, which have been previously implicated in atherosclerotic inflammation [23,45]. ENA-78 has been shown to be chemotactic for neutrophils and stimulate neutrophilic degranulation causing release of myeloperoxidase and generating reactive oxygen species [24,25]. In addition, ENA-78 is involved in platelet-dependent activation of monocytes, displays angiogenic properties, and has been implicated in diseases such as obesity, diabetes, subclinical atherosclerosis, acute coronary syndromes, ischemic stroke, abdominal aortic aneurysm, and thrombosis [27,28,32,44,46-51]. Hypertension is a risk factor for adverse events such as atherosclerosis, stroke, and abdominal aortic aneurysm, and ENA-78 is overexpressed in these situations. We have shown *CXCL5* polymorphisms to be associated with ENA-78 concentrations, blood pressure, and prognosis following acute coronary syndromes [27,33]. Thus, the role of *CXCL5* in CVD should be further explored. As final hypothesis-generating evidence of a link between the *CXCL5* pathway and blood pressure, statins have been hypothesized to have mild antihypertensive effects, and we have shown that atorvastatin reduces ENA-78 production from human endothelial cells in a dose-dependent fashion [52,53]. Our findings, along with existing data, support the need for future investigation of *CXCL5* as a hypertension- and CVD-susceptibility gene.

## Competing interests

The authors declare that they have no competing interests.

## Authors’ contributions

ALB performed statistical analyses and drafted the manuscript. CLA enrolled the study subjects and drafted the manuscript. HA assisted in the molecular genetic studies and provided critical revision of the manuscript. TYL assisted in the molecular genetic studies. GJW assisted in the molecular genetic studies. RSS assisted with the clinical study. IZ conceived the manuscript, enrolled the study subjects, and drafted the manuscript. All authors read and approved the final manuscript.
